# On the Performance of Cognitive Satellite-Terrestrial Relay Networks with Channel Estimation Error and Hardware Impairments

**DOI:** 10.3390/s18103292

**Published:** 2018-09-30

**Authors:** Kefeng Guo, Kang An, Bangning Zhang, Yuzhen Huang, Daoxing Guo

**Affiliations:** 1 College of Communication Engineering, Army Engineering University of PLA(Its Old Name Is PLA University of Science and Technology), Nanjing 210007, China; guokefeng.cool@163.com (K.G.); bangning_zhang@sina.com (B.Z.); 2 National University of Defense Technology, Nanjing 210016, China; ankang@nuaa.edu.cn; 3 Artificial Intelligence Research Center, National Innovation Institute of Defense Technology, Beijing 100091, China; yzh_huang@sina.com

**Keywords:** cognitive satellite-terrestrial relay networks, multiple primary users, hardware impairments (HIs), channel estimation error (CSI)

## Abstract

This paper investigates the joint impact of channel estimation errors (CEEs) and hardware impairments (HIs) on the performance of a cognitive satellite-terrestrial relay network (CSTRN), where the terrestrial and satellite links are considered following Rayleigh fading and shadowed Rician (SR) fading distributions, respectively. Besides, the terrestrial relay is working in half-duplex decode-and-forward (DF) mode. By employing a general and practical model to account for both the CEEs and HIs at each link, the end-to-end signal-to-noise-plus-distortion-and-error ratio (SNDER) is first obtained for the CSTRN. Then, closed-form expressions for the outage probability (OP) and throughput of the CSTRN are obtained, which allows us to demonstrate the aggregate impact of CEEs and HIs. In order to gain insightful findings, we further elaborate on the asymptotic OP and throughput at the high signal-to-noise-ratio (SNR) condition and quantitatively determine the fundamental performance ceiling. Finally, Monte Carlo (MC) computer simulations are provided to verify the correctness of the analytical results. Besides, with representative numerical analysis’s help, interesting findings are presented.

## 1. Introduction

The target of next generation wireless communication networks contains many connected devices, data rates in the range of Gbps, increased reliability, lower latencies, environmentally-friendly and improved coverage, energy-efficiency and low-cost operation [1]. Satellite mobile communication has drawn extensive interest in radio technology research, which aims to offer enough overlap with ordinary industrial facilities as a result of the performance limits in the existing cellular spectrum and the growing interest in the exploration of supplementary resource [2]. In this regard, researchers have recently envisaged the integration of satellite and terrestrial systems to form a satellite-terrestrial relay network (STRN) architecture [3,4], which basically implants terrestrial relay cooperation into satellite mobile communications. Such STRN can provide broadcast/multicast services and uninterrupted coverage to portable and mobile users. Among the existing works, the authors of [3] derived the asymptotic results for the STRN in the presence of the amplify and forward relaying protocol with fixed gain. In [4], the outage probability (OP) of STRN was studied in the presence of multiple users with opportunistic user scheduling. In [5], the authors examined the problem of amplify-and-forward (AF)-based relaying in a hybrid satellite-terrestrial link and derived the novel expressions for the symbol error rate (SER) of the considered system. In [6], the authors investigated the performance of integrated wireless sensor and multi-beam satellite networks under terrestrial interference and derived the closed-form approximations of capacity per beam. In [7], the authors proposed the energy-efficient optimal power allocation schemes in the cognitive satellite-terrestrial networks for non-real-time and real-time applications and maximized the energy efficiency of the cognitive satellite user. In [8], the OP was studied for the multiple terrestrial STRN with the switch-and-stay combining scheme in the presence of HIs. In [9], the authors studied the OP of the STRN in the presence of multi-antenna multiple users. Besides, the outdated channel state information (CSI) and co-channel interference were both considered in the system.

Cognitive radio is considered as an important technology that is regarded as a method of spectrum management in the usual wireless communication system due to the reason that primary and secondary networks coexist by utilizing identical resources [10,11,12]. The fusion of the cognitive satellite-terrestrial relay network (CSTRN) has been extensively investigated in both academic and industry areas. In [10], the authors minimized the transmit power by optimizing the artificial noise and cooperative beamforming in the CSTRN. In [11], the authors investigated the impact of multiple primary users and fading on the spectrum sensing of a classical energy detector. In [12], to minimize the transmit power and satisfy the outage in the CSTRN, a beamforming method was proposed. The authors in [13] explored the possibility of maximizing spectrum efficiency for satellite-terrestrial uplink transmissions. Moreover, in [14], the main aspects of the CSTRN were studied, and possible practical scenarios for the CSTRN were presented. In [15], a mathematic approach was provided to achieve higher efficiency of the OP for the CSTRN. In [16], the authors investigated the OP of the CSTRN in the presence of multiple secondary networks.

To its detriment, the hardware of the aforementioned works is assumed to be perfect, which cannot be realized in practical systems. In fact, the transceiver node in wireless networks often suffers several types of hardware impairments (HIs) such as phase noise (PN), high power amplifier (HPA) non-linearities and in-phase quadrature-phase imbalance (IQI) in the oscillator [17]. Due to these reasons, the wanted transmit signal and practical emitted signal will be mismatched as a result of distortions for the received signal during the transmission course [18]. In [19], the authors studied the impact of HIs and fading on the spectrum sensing performance of an energy detector full-duplex wireless system.

So far, the performance of satellite relay dual-hop networks with HIs has been investigated in [20]. The effects of HIs and co-channel interference on the STRN were first considered in [21]. In [22], the authors studied the individual impact of HIs on the CSTRN.

However, it should be pointed out that most of the prior works considered the ideal CSI in the CSTRN. In practice, due to the high delay and fast fading natures, it is very hard to get the accurate CSI of the satellite links; thus, channel estimation is usually needed for the satellite links; however, channel estimation errors (CEEs) always exist when using the channel estimation. Besides, owing to the time-varying character of terrestrial links, the availability of the perfect CSI cannot be obtained in practice, either. Currently, the issue of CEEs for the CSTRN has been studied in several works [23,24]. In [23], the impact of non-ideal CSI for both the harmful terrestrial interference link and desired satellite link on the power control scheme was investigated in the CSTRN. In [24], the authors analyzed the secrecy performance of the primary satellite system in the CSTRN with the underlay scheme and imperfect CSI. To the authors’ best knowledge, the joint impact of CEEs and HIs on the performance of the CSTRN remains unreported, which leads to the contribution given in this paper.

In this paper, we focus on the performance limitations in a CSTRN with both HIs and CEEs, where the primary terrestrial system shares the spectral resources with the secondary satellite system. Particularly, our main contributions can be outlined as follows:

- We first establish a general and practical framework of a CSTRN with HIs and CEEs, where the cognitive satellite networks coexists with the multiple user primary terrestrial network with the interference temperature constraints.
- After obtaining the end-to-end signal-to-noise-plus-distortion-and-error ratio (SNDER), novel closed-form OP and throughput expressions of the considered networks are obtained, which give a general and applicable method to characterize the key parameters on the considered network.
- To gain more insights, the asymptotic analysis of OP and throughput at high signal-to-noise-ratios (SNRs) are provided, which enable a quantitative characterization of the impact of HI levels and CSI imperfections on the considered network.

Notations: Bold uppercase letters denote matrices, and bold lowercase letters denote vectors; $\left|\cdot \right|$ is the absolute value of a complex scalar; $\exp\left(\cdot \right)$ is the exponential function, $E\left[\cdot \right]$ the expectation operator, $CN\left(a,b\right)$ the complex Gaussian distribution, where a is the mean value of random complex vector and *b* the covariance matrix, and $B\left(.,.\right)$ denotes the Beta function.

## 2. System Model and Problem Formulation

**Figure 1:**
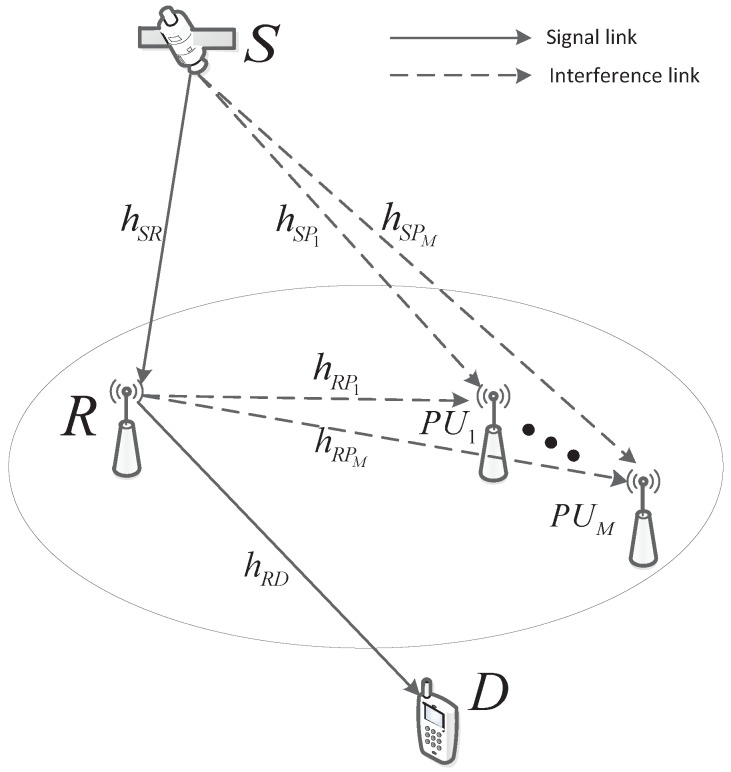
Illustration of the system model.

As illustrated in Figure 1, in this paper, the CSTRN, which has one source (S), one secondary destination (D), a terrestrial relay (R) and *M* terrestrial primary users (PUs), is considered. All of them are equipped with a single antenna. Furthermore, all nodes in the CSTRN are assumed suffering from HIs. S and D can communicate with each other only via R due to fog, rain, haze or heavy shadowing [25]. One R with the half-duplex decode-and-forward (DF) protocol is applied in the network, and the whole transmission occurs in two time slots.

Through the first time slot, the signal $s\left(t\right)$ with $E\left[{\left|s\left(t\right)\right|}^{2}\right]=1$ will be transmitted from S to R; hence, the signal received at R is given by:

(1) $$\begin{array}{c} y_{R}\left(t\right)=h_{SR}\left[\sqrt{P_{S}}s\left(t\right)+\eta_{1}\left(t\right)\right]+n_{R}\left(t\right), \end{array}$$

where $h_{SR}$ represents the complex channel coefficient between S and R whose absolute value follows the shadowed Rician (SR) fading. $P_{S}$ denotes the transmit power from S; $\eta_{1}\left(t\right)$ presents the distortion noise owing to HIs, which can be shown as $\eta_{1}\left(t\right)∼CN\left(0,k_{1}^{2}P_{S}\right)$; $k_{1}$ is the error vector magnitude (EVM), which indicates the extent of the non-ideality of the hardware [26]. $n_{R}\left(t\right)$ is the additive white Gaussian noise (AWGN) at R, which can be distributed as $CN\left(0,\delta_{R}^{2}\right)$.

During the second time slot, the signal received will be transmitted to D by R via the DF protocol. The signal received at D is presented as:

(2) $$\begin{array}{c} y_{D}\left(t\right)=h_{RD}\left[\sqrt{P_{R}}s\left(t\right)+\eta_{2}\left(t\right)\right]+n_{D}\left(t\right), \end{array}$$

where $h_{RD}$ denotes the complex channel coefficient between R and D whose absolute value follows Rayleigh fading, $P_{R}$ is the power of R, $\eta_{2}\left(t\right)$ is the distortion noise caused by HIs, which has the form as $\eta_{2}\left(t\right)∼CN\left(0,k_{2}^{2}P_{R}\right)$ and $k_{2}$ quantifies the level of impairments and is measured experimentally as EVM. $n_{D}\left(t\right)$ is the AWGN at D presented as $n_{D}∼CN\left(0,\delta_{D}^{2}\right)$.

Now, as pointed out earlier, only non-ideal CSI of both satellite and terrestrial links is known; thus, non-ideal channel gains need to be found before the performance evaluations. Hence, the channel can be modeled as [23,24] (we should note that this model has been proven and extensively used in existing works such as [23,24]),

(3) $$\begin{array}{c} h_{X}={\tilde{h}}_{X}+e_{h_{X}},X\in \left\{SR,RP,SP,RP\right\}, \end{array}$$

where $h_{X}$ and ${\tilde{h}}_{X}$ represent the practical and estimated fading coefficients, respectively, $h_{X}$ and ${\tilde{h}}_{X}$ are assumed to have combined ergodicity and $e_{h_{X}}$ denotes the estimation error, which is orthogonal to the channel estimate coefficient ${\tilde{h}}_{X}$ with modeling as a zero mean complex Gaussian distribution [23] with its variance:

(4) $$\begin{array}{c} {\bar{\Upsilon }}_{h_{X}}=E\left\{{\left|h_{X}\right|}^{2}\right\}-E\left\{{\left|{\tilde{h}}_{X}\right|}^{2}\right\}=\frac{1}{T_{X}{\bar{\Upsilon }}_{X}+1}, \end{array}$$

where $T_{X}$ is the length of training symbols and ${\bar{\Upsilon }}_{X}=E\left\{\Upsilon_{X}\right\}=\frac{P_{X}E\left\{{\left|h_{X}\right|}^{2}\right\}}{N_{X}}$ is the mean SNR of the training symbol for the transmitting link with HIs. Besides $P_{X}=\left(1-\sigma \right)P_{total}$ with the scale coefficient $\sigma \in \left(0,1\right)$, $P_{X}$ presents the power of the pilot symbols, $P_{total}$ the total transmission power and $N_{X}$ the AWGN variance of the transmitting links. Since the detection and estimation of training signals are also impacted by HIs, ${\bar{\Upsilon }}_{X}$ can be rewritten with the help of the training symbols’ SNR for the system that has ideal hardware ${\bar{\Upsilon }}_{idX}$ as:

(5) $$\begin{array}{c} {\bar{\Upsilon }}_{X}=\frac{{\bar{\Upsilon }}_{idX}}{{\bar{\Upsilon }}_{idX}k_{X}^{2}+1}, \end{array}$$

where $k_{X}$ presents the EVM parameters, which influence the training symbols of the transmitting links. Then, substituting Equation (5) into Equation (4), we can obtain:

(6) $$\begin{array}{c} {\bar{\Upsilon }}_{h_{X}}=\frac{{\bar{\Upsilon }}_{idX}k_{X}^{2}+1}{\left(T_{X}+k_{X}^{2}\right){\bar{\Upsilon }}_{idX}+1}, \end{array}$$

where ${\bar{\Upsilon }}_{h_{X}}$ reports the accuracy of channel estimation and is presented as the minimum mean square error (MMSE).

Further, in the CSTRN with CEEs and HIs, to limit the interference power at PUs below a pre-determined threshold *Q*, the instantaneous powers at S and R should be constrained as:

$$\begin{array}{c} E\left\{\sum_{i=1}^{M}{\left|\left({\tilde{h}}_{SP_{i}}+e_{h_{SP_{i}}}\right)\left(s\left(t\right)+\eta_{SP_{i}}\left(t\right)\right)\right|}^{2}\right\}\leq Q,\\ E\left\{\sum_{i=1}^{M}{\left|\left({\tilde{h}}_{RP_{i}}+e_{h_{RP_{i}}}\right)\left(s\left(t\right)+\eta_{RP_{i}}\left(t\right)\right)\right|}^{2}\right\}\leq Q, \end{array}$$

where $\eta_{SP_{i}}\left(t\right)∼CN\left(0,k_{SP_{i}}^{2}P_{S}\right)$ and $\eta_{RP_{i}}\left(t\right)∼CN\left(0,k_{RP_{i}}^{2}P_{R}\right)$ are the distortion noise components caused by HIs in transmitting processing at S and R and $k_{SP_{i}}$ and $k_{RP_{i}}$ represent the HI level at PUs. Consequently, we have:

(7) $$\begin{array}{c} P_{S}=\frac{Q}{\sum_{i=1}^{M}{\left|\left({\left|{\tilde{h}}_{SP_{i}}\right|}^{2}+{\bar{\Upsilon }}_{h_{SP_{i}}}\right)\left(1+k_{SP_{i}}^{2}\right)\right|}^{2}}, \end{array}$$

(8) $$\begin{array}{cc} P_{R} & =\frac{Q}{\sum_{i=1}^{M}{\left|\left({\left|{\tilde{h}}_{RP_{i}}\right|}^{2}+{\bar{\Upsilon }}_{h_{RP_{i}}}\right)\left(1+k_{RP_{i}}^{2}\right)\right|}^{2}}. \end{array}$$

Now, it has assumed that at S and R, the maximum transmit power is large enough and can be neglected to meet the interference constraint [27].

Then, from Equations (1) and (7), the final SNDER of $y_{R}\left(t\right)$ is given by:

(9) $$\begin{array}{c} \gamma_{R}=\frac{\gamma_{SR}Q}{\gamma_{SR}Qk_{1}^{2}+\delta_{R}^{2}\gamma_{SP}\left(1+k_{SP}^{2}\right)\sigma^{-1}+A}, \end{array}$$

where $\gamma_{SR}=\frac{\sigma Q}{\delta_{R}^{2}}{\left|{\tilde{h}}_{SR}\right|}^{2}$, $\gamma_{SP}=\frac{\sigma Q}{\delta_{R}^{2}}\sum_{i=1}^{M}{\left|{\tilde{h}}_{SP_{i}}\right|}^{2}$, $A=\frac{\sigma Q^{2}}{\delta_{R}^{2}}{\bar{\Upsilon }}_{h_{SR}}\left(1+k_{1}^{2}\right)+Q\sum_{i=1}^{M}{\bar{\Upsilon }}_{h_{SP_{i}}}\left(1+k_{SP}^{2}\right)$ with the assumption $k_{SP_{1}}=\cdots =k_{SP_{i}}=\cdots =k_{SP_{M}}=k_{SP}$.

In the same manner, the final SNDER at D is given by:

(10) $$\begin{array}{c} \gamma_{D}=\frac{\gamma_{RD}Q}{\gamma_{RD}Qk_{2}^{2}+\delta_{D}^{2}\gamma_{RP}\left(1+k_{RP}^{2}\right)\sigma^{-1}+B}, \end{array}$$

where $\gamma_{RD}=\frac{\sigma Q}{\delta_{D}^{2}}{\left|{\tilde{h}}_{RD}\right|}^{2}$, $\gamma_{RP}=\frac{\sigma Q}{\delta_{D}^{2}}\sum_{i=1}^{M}{\left|{\tilde{h}}_{RP_{i}}\right|}^{2}$, $B=\frac{Q^{2}\sigma }{\delta_{D}^{2}}{\bar{\Upsilon }}_{h_{RD}}\left(1+k_{2}^{2}\right)+Q\sum_{i=1}^{M}{\bar{\Upsilon }}_{h_{RP_{i}}}\left(1+k_{RP}^{2}\right)$ and $k_{RP_{1}}=k_{RP_{2}}=\cdots k_{RP_{M}}=k_{RP}$.

As discussed before, R forwards the signal received with the DF protocol, and the received SNDER of the system is given by:

(11) $$\begin{array}{c} \gamma_{e}=\min\left(\gamma_{R},\gamma_{D}\right). \end{array}$$

**Remark** **1.** *It should be mentioned that we establish a more general framework of the CSTRN by taking multiple PUs, CEEs and HIs into consideration, where the propagation factors of path loss, channel shadowing conditions and satellite beam pattern are also included. Specifically, our paper consists of the system model in [4] as a special case, where only a single PU, perfect hardware and accurate CSI are assumed. Furthermore, our work extends the works in [22,23] as a special case where only HIs and CEEs were considered, respectively.*

## 3. Performance Analysis

### 3.1. Preliminary Results

#### 3.1.1. Terrestrial Channel Model

In this paper, we suppose that all of the terrestrial links undergo independent and identically distribution (i.i.d) Rayleigh fading.

Next, according to [21], the PDF for $\gamma_{RP}$ and the CDF of $\gamma_{RD}$ are given, respectively, by:

(12) $$\begin{array}{c} f_{\gamma_{RP}}\left(x\right)=\sum_{i=1}^{\rho \left(A_{RP}\right)}\sum_{j=1}^{\tau_{i}\left(A_{RP}\right)}\chi_{i,j}\left(A_{RP}\right)\frac{\mu_{\langle i\rangle }^{-j}}{\left(j-1\right)!}x^{j-1}e^{-x/\mu_{\langle i\rangle }}, \end{array}$$

(13) $$\begin{array}{c} F_{\gamma_{RD}}\left(x\right)=1-e^{-\frac{x}{{\bar{\gamma }}_{RD}}}, \end{array}$$

where $A_{RP}=\mathrm{diag}\left(\mu_{1},\mu_{1},\ldots ,\mu_{M}\right)$, $\rho \left(A_{RP}\right)$ is the number of distinct diagonal elements of $A_{RP}$, $\mu_{\langle 1\rangle }>\mu_{\langle 2\rangle }>\ldots >\mu_{\langle \rho \left(A_{RP}\right)\rangle }$ are the ascending order of $\mu_{\langle i\rangle }$, $\tau_{i}\left(A_{RP}\right)$ is the multiplicity of $\mu_{\langle i\rangle }$ and $\chi_{i,j}\left(A_{RP}\right)$ is the (i,j)-th characteristic coefficient of $A_{RP}$ [21].

#### 3.1.2. Satellite Channel Model

In modern satellite communications, multibeam technology is widely used to increase the spectral efficiency, which should be taken into account in modeling the satellite channel. For a geosynchronous Earth orbit (GEO) satellite, multiple beams are often generated through array-fed reflectors, which is more efficient than direct radiating arrays. In this case, the radiation pattern of each beam is fixed, so that the on-board precessing can be significantly reduced [28]. Furthermore, the time division multiple access (TDMA) scheme is adopted so that there is only one Earth station (ES) scheduled within each beam at any given time.

Next, the channel coefficient ${\tilde{h}}_{SJ}$ between the ES and the *k*-th on-board beam for the downlink is given by:

(14) $$\begin{array}{c} {\tilde{h}}_{SJ}=C_{SJ}g_{SJ},\left(J\in \left\{R,P_{i}\right\}\right), \end{array}$$

where $g_{SJ}$ represents the complex coefficient of the satellite channel and $C_{SJ}$ denotes the radio propagation loss including the effects of free space loss (FSL) and the antenna pattern, which is described as:

(15) $$\begin{array}{c} C_{SJ}=\frac{\lambda }{4\pi }\frac{\sqrt{G_{SJ}G_{ES}}}{\sqrt{d^{2}+d_{0}^{2}}}, \end{array}$$

where λ denotes the carrier wavelength, *d* is the distance between the ES and the center of the *k*-th center beam and $d_{0}\approx$ 35,786 km is the height of a GEO satellite. Besides, $G_{ES}$ is the antenna gain of the ES and $G_{SJ}$ is the *k*-th satellite on-board beam gain.

According to [29], the antenna gain for the ES with parabolic antenna can be approximately expressed as:

(16) $$\begin{array}{c} G_{ES}\left(dB\right)≃\left\{\begin{array}{cc} {\bar{G}}_{\max}, & for\;0^{\circ }<\beta <1^{\circ }\\ 32-25\log\beta , & for1^{\circ }<\beta <48^{\circ }\\ -10, & for\;48^{\circ }<\beta \leq 180^{\circ }, \end{array}\right. \end{array}$$

where ${\bar{G}}_{\max}$ is the maximum beam gain at the boresight and β the off-boresight angle. As for $G_{SJ}$, by defining $\theta_{k}$ as the angle between the ES position and the *k*-th beam center with respect to the satellite and ${\bar{\theta }}_{k}$ as the 3-dB angle of the *k*-th on-board beam, the antenna gain from the *k*-th beam to the ES is approximated by [30]:

(17) $$\begin{array}{c} G_{SJ}≃G_{\max}{\left(\frac{J_{1}\left(u_{k}\right)}{2u_{k}}+36\frac{J_{3}\left(u_{k}\right)}{u_{k}^{3}}\right)}^{2}, \end{array}$$

where $G_{max}$ denotes the maximal beam gain, $u_{k}=2.07123\sin\theta_{k}/\sin{\bar{\theta }}_{k}$ and $J_{1}$ and $J_{3}$ denote the first-kind Bessel function of order one and three, respectively. In order to obtain the maximum beam gain, hence $\theta_{k}\to 0$, as a result of $G_{SJ}\approx G_{\max}$. On this foundation, we have ${\tilde{h}}_{SJ}=C_{SJ}^{\max}g_{SJ}$ ($C_{SJ}^{max}$ can be derived by submitting (17) and (16) into (15) when $\theta_{k}\to 0$, which is given by $C_{SJ}^{\max}\approx \frac{\lambda }{4\pi }\frac{\sqrt{G_{\max}G_{ES}}}{\sqrt{d^{2}+d_{0}^{2}}}$).

As for the complex random shadowing $g_{SJ}$, besides the mathematical models, including Loo, Barts–Stutzman and Karasawa, the SR channel proposed in [31] is the commonly-used channel model for LMS communication [3,4,32,33].

According to [31], the complex random shadowing of $g_{SJ}$ undergoing the SR model can be expressed as:

(18) $$\begin{array}{c} g_{SJ}=A\exp\left(j\vartheta \right)+Z\exp\left(j\psi \right), \end{array}$$

where *A* and *Z* denote the amplitudes of the scattering and the LOS components, which are the independent stationary random processes following Rayleigh and Nakagami-*m* distributions, respectively. Besides, ϑ is a stationary random phase uniformly distributed over $\left[0,2\pi \right)$, and ψ is the deterministic phase of the LOS component. Furthermore, the PDF of $\gamma_{SJ}={\bar{\gamma }}_{SJ}{\left|C_{SJ}^{\max}g_{SJ}\right|}^{2}$ is given by:

(19) $$\begin{array}{c} f_{\gamma_{SJ}}\left(x\right)=\frac{\alpha_{SJ}}{{\bar{\gamma }}_{SJ}}{e^{-\frac{\beta_{SJ}}{{\bar{\gamma }}_{SJ}}}}_{1}F_{1}\left(m_{SJ};1;\frac{\delta_{SJ}}{{\bar{\gamma }}_{SJ}}x\right),x>0, \end{array}$$

where ${}_{1}F_{1}\left(a;b;x\right)$ denotes the confluent hypergeometric function defined in [34]. ${\bar{\gamma }}_{SJ}$ is the average SNR between Alice and the ξ-th R with expression as ${\bar{\gamma }}_{SJ}=\frac{\delta Q}{\delta_{R}^{2}}$, $\alpha_{SJ}={\left(\frac{2b_{SJ}m_{SJ}}{2b_{SJ}m_{SJ}+\Omega_{SJ}}\right)}^{m_{SJ}}/2b_{SJ}$, $\beta_{SJ}=\frac{1}{2b_{SJ}}$, $\delta_{SJ}=\frac{\Omega_{SJ}}{2b_{SJ}\left(2b_{SJ}m_{SJ}+\Omega_{SJ}\right)}$ with $\Omega_{SJ}$, $2b_{SJ}$ and $m_{SJ}\geq 0$ being the average power of the LOS component, the average power of the multipath component and the fading severity parameter ranging from 0–*∞*, respectively. By considering *m* being an integer, the PDF of $\gamma_{SJ}$ is given by:

(20) $$\begin{array}{c} f_{\gamma_{SJ}}\left(x\right)=\alpha \sum_{k=0}^{m_{SJ}-1}\frac{{\left(1-m_{SJ}\right)}_{k}{\left(-\delta_{SJ}\right)}^{k}}{{\left(k!\right)}^{2}{\left({\bar{\gamma }}_{SJ}\right)}^{k+1}}x^{k}\exp\left(-\Delta_{SJ}x\right), \end{array}$$

where $\Delta_{SJ}=\frac{\beta_{SJ}-\delta_{SJ}}{{\bar{\gamma }}_{SJ}}$ and ${\left(\cdot \right)}_{k}$ is the Pochhammer symbol.

Hence, the CDF of $\gamma_{SJ}$ is given by:

(21) $$\begin{array}{c} F_{\gamma_{SJ}}\left(x\right)=1-\alpha \sum_{k=0}^{m_{SJ}-1}\sum_{t=0}^{k}\frac{{\left(1-m_{SJ}\right)}_{k}{\left(-\delta_{SJ}\right)}^{k}}{k!{\left({\bar{\gamma }}_{SJ}\right)}^{k+1}t!\Delta_{SJ}^{k-t+1}}x^{t}e^{-\Delta_{SJ}x}. \end{array}$$

In the manuscript, we consider the worst case (The worst case is for the secondary users. In this paper, it means that all the primary users operated on the same frequency spectrum and worked together; hence, the SNR is the sum of the SNR of all primary users. On this foundation, the left available spectrum is minimal, which is the worst case for the secondary user.); hence:

(22) $$\begin{array}{c} \gamma_{SP}=\sum_{i=1}^{M}\gamma_{SP_{i}}. \end{array}$$

From [24], the probability distribution function (PDF) of $\gamma_{SP}$ is given by:

(23) $$\begin{array}{c} f_{\gamma_{SP}}\left(x\right)=\sum_{\xi_{1}=0}^{m_{SP}-1}\cdots \sum_{\xi_{M}=0}^{m_{SP}-1}\Xi \left(M\right)x^{\Lambda_{SP}-1}e^{-\Delta_{SP}x}, \end{array}$$

where:

$$\Xi \left(M\right)\overset{\Delta }{=}\prod_{\tau =1}^{M}\zeta \left(\xi_{\tau }\right)\alpha_{SP}^{M}\prod_{\upsilon =1}^{M-1}B\left(\sum_{l=1}^{\upsilon }\xi_{l}+\upsilon ,\xi_{\upsilon +1}+1\right),$$

$\Lambda_{SP}\overset{\Delta }{=}\sum_{\tau =1}^{M}\xi_{\tau }+M$, $\zeta \left(\xi_{\tau }\right)=\frac{{\left(1-m_{SP}\right)}_{\xi_{\tau }}{\left(-\delta_{SP}\right)}^{\xi_{\tau }}}{{\left(\xi_{\tau }!\right)}^{2}{\left({\bar{\gamma }}_{SP}\right)}^{\xi_{\tau }+1}}$, $\Delta_{SP}=\frac{\beta_{SP}-\delta_{SP}}{{\bar{\gamma }}_{SP}}$, $\alpha_{SP}\overset{\Delta }{=}\frac{{\left(\frac{2b_{SP}m_{SP}}{2b_{SP}m_{SP}+\Omega_{SP}}\right)}^{m_{SP}}}{2b_{SP}}$, $\beta_{SP}\overset{\Delta }{=}\frac{1}{2b_{SP}}$,

$\delta_{SP}\overset{\Delta }{=}\frac{\Omega_{SP}}{2b_{SP}\left(2b_{SP}m_{SP}+\Omega_{SP}\right)}$ and $B\left(.,.\right)$ denotes the Beta function [34].

### 3.2. OP

According to [18], OP is an important performance measurement, which is known as the SNDER falls below a predefined threshold $\gamma_{0}$, namely:

(24) $$\begin{array}{c} P_{out}\left(\gamma_{0}\right)=\mathrm{Pr}\left(\gamma_{R}\leq \gamma_{0}\right)+\mathrm{Pr}\left(\gamma_{D}\leq \gamma_{0}\right)-\mathrm{Pr}\left(\gamma_{R}\leq \gamma_{0}\right)\mathrm{Pr}\left(\gamma_{D}\leq \gamma_{0}\right). \end{array}$$

In what follows, $\mathrm{Pr}\left(\gamma_{R}\leq \gamma_{0}\right)$ and $\mathrm{Pr}\left(\gamma_{D}\leq \gamma_{0}\right)$ will be given, respectively.

Firstly, we get the closed-form expression of $\mathrm{Pr}\left(\gamma_{R}\leq \gamma_{0}\right)$. From Equation (9), when $\gamma_{0}<1/k_{1}^{2}$, we have:

(25) $$\begin{array}{cc} \mathrm{Pr}\left(\gamma_{R}\leq \gamma_{0}\right) & =\mathrm{Pr}\left(\frac{\gamma_{SR}Q}{\gamma_{SR}Qk_{SR}^{2}+\gamma_{SP}\left(1+k_{SP}^{2}\right)\delta_{R}^{2}}\leq \gamma_{0}\right)\\ & =\int_{0}^{\infty }\int_{0}^{C_{2}y+C_{3}}f_{\gamma_{SR}}\left(x\right)f_{\gamma_{SP}}\left(y\right)dxdy\\ & =\int_{0}^{\infty }F_{\gamma_{SR}}\left(yC_{2}+C_{3}\right)f_{\gamma_{SP}}\left(y\right)dy, \end{array}$$

where $C_{2}=\frac{\delta_{R}^{2}\left(1+k_{SP}^{2}\right)\gamma_{0}}{\sigma Q\left(1-k_{1}^{2}\gamma_{0}\right)}$ and $C_{3}=\frac{A\gamma_{0}}{Q\left(1-k_{1}^{2}\gamma_{0}\right)}$.

By taking Equations (20) and (23) into Equation (25), we can get Equation (26), which is shown at the top of this page.

(26) PrγR≤γ0=∑ξ1=0mSP−1⋯∑ξM=0mSP−1ΞMΛSP−1!ΔSP−ΛSP−∑t=1mSR−1∑s=0tαSRξtt!γ¯SRt+1s!ΔSRt−s+1∑v=0ssvC3s−vC2ve−ΔSRC3ΛSP−1+v!C2ΔSR+ΔSP−ΛSP−v=1−∑ξ1=0mSP−1⋯∑ξM=0mSP−1ΞM∑t=1mSR−1∑s=0tαSRξtt!γ¯SRt+1s!ΔSRt−s+1∑v=0ssvC3s−vC2ve−ΔSRC3ΛSP−1+v!C2ΔSR+ΔSP−ΛSP−v.

Then, with similar manners, when $\gamma_{0}<1/k_{2}^{2}$, $\mathrm{Pr}\left(\gamma_{D}\leq \gamma_{0}\right)$ is given by:

(27) $$\begin{array}{cc} \mathrm{Pr}\left(\gamma_{D}\leq \gamma_{0}\right) & =\mathrm{Pr}\left(\frac{\gamma_{RD}Q}{\gamma_{RD}Qk_{2}^{2}+\delta_{D}^{2}\gamma_{RP}\left(1+k_{RP}^{2}\right)+B}\leq \gamma_{0}\right)\\ & =\int_{0}^{\infty }\int_{0}^{yD_{1}+D_{2}}f_{\gamma_{RD}}\left(x\right)f_{\gamma_{RP}}\left(y\right)dxdy\\ & =\int_{0}^{\infty }F_{\gamma_{RD}}\left(yD_{1}+D_{2}\right)f_{\gamma_{RP}}\left(y\right)dy, \end{array}$$

where $D_{1}=\frac{\delta_{D}^{2}\left(1+k_{RP}^{2}\right)\gamma_{0}}{\sigma \left(1-k_{2}^{2}\gamma_{0}\right)Q}$ and $D_{2}=\frac{B\gamma_{0}}{\left(1-k_{2}^{2}\gamma_{0}\right)Q}$.

Then, substituting Equations (12) and (13) into Equation (27), we can obtain Equation (28), which is presented at the top of this page.

(28) $$\begin{array}{cc} \mathrm{Pr}\left(\gamma_{D}\leq \gamma_{0}\right) & =\sum_{i=1}^{\rho \left(A_{RP}\right)}\sum_{j=1}^{\tau_{i}\left(A_{RP}\right)}\chi_{i,j}\left(A_{RP}\right)-\sum_{i=1}^{\rho \left(A_{RP}\right)}\sum_{j=1}^{\tau_{i}\left(A_{RP}\right)}\frac{\chi_{i,j}\left(A_{RP}\right)\mu_{\langle i\rangle }^{-j}}{{\left(1/\mu_{\langle i\rangle }+D_{2}/{\bar{\gamma }}_{RD}\right)}^{j}}\\ & =1-\sum_{i=1}^{\rho \left(A_{RP}\right)}\sum_{j=1}^{\tau_{i}\left(A_{RP}\right)}\frac{\chi_{i,j}\left(A_{RP}\right)\mu_{\langle i\rangle }^{-j}}{{\left(1/\mu_{\langle i\rangle }+D_{2}/{\bar{\gamma }}_{RD}\right)}^{j}}. \end{array}$$

Finally, by substituting Equations (26) and (28) into Equation (24), the final expression of OP is obtained as:

(29) Poutγ0=∑ξ1=0mSP−1⋯∑ξM=0mSP−1ΞMΛSP−1!ΔSP−ΛSP−∑t=1mSR−1∑s=0tαSRξtt!γ¯SRt+1s!ΔSRt−s+1∑v=0ssvC3s−vC2ve−ΔSRC3ΛSP−1+v!C2ΔSR+ΔSP−ΛSP−v+∑i=1ρARP∑j=1τiARPχi,jARP−∑i=1ρARP∑j=1τiARPχi,jARPμi−j1/μi+D2/γ¯RDj−∑ξ1=0mSP−1⋯∑ξM=0mSP−1ΞMΛSP−1!ΔSP−ΛSP−∑t=1mSR−1∑s=0tαSRξtt!γ¯SRt+1s!ΔSRt−s+1∑v=0ssvC3s−vC2ve−ΔSRC3ΛSP−1+v!C2ΔSR+ΔSP−ΛSP−v×∑i=1ρARP∑j=1τiARPχi,jARP−∑i=1ρARP∑j=1τiARPχi,jARPμi−j1/μi+D2/γ¯RDj,γ0<min1k12,1k221,γ0≥min1k12,1k22.

After some simplifications, Equation (29) can be rewritten as:

(30) Poutγ0=1−∑i=1ρARP∑j=1τiARP∑ξ1=0mSP−1⋯∑ξM=0mSP−1∑t=1mSR−1∑s=0tΞMχi,jARPμi−j1/μi+D2/γ¯RDj×αSRξtt!γ¯SRt+1s!ΔSRt−s+1∑v=0ssvC3s−vC2ve−ΔSRC3ΛSP−1+v!C2ΔSR+ΔSP−ΛSP−v,γ0<min1k12,1k221,γ0≥min1k12,1k22.

### 3.3. Asymptotic OP

Recalling the fact in Equation (20), when ${\bar{\gamma }}_{SR}$ is large enough, it can be presented as:

(31) $$\begin{array}{c} F_{\gamma_{SR}}\left(x\right)\approx \frac{\alpha_{SR}x}{{\bar{\gamma }}_{SR}}. \end{array}$$

Taking Equations (23) and (31) into Equation (25), we could have Equation (32), which is shown at the top of this page.

(32) $$\begin{array}{c} \mathrm{Pr}\left(\gamma_{R}\leq \gamma_{0}\right)\approx \sum_{\xi_{1}=0}^{m_{SP}-1}\cdots \sum_{\xi_{M}=0}^{m_{SP}-1}\frac{\alpha_{SR}\Xi \left(M\right)}{{\bar{\gamma }}_{SR}}\left[\frac{C_{1}\Lambda_{SP}!}{\Delta_{SP}^{\Lambda_{SP}+1}}+\frac{C_{2}\left(\Lambda_{SP}-1\right)!}{\Delta_{SP}^{\Lambda_{SP}}}\right]. \end{array}$$

Utilizing the similar method and supposing the identical channel parameters, the CDF of $\gamma_{RD}$ at high SNRs is given by:

(33) $$\begin{array}{c} F_{\gamma_{RD}}\left(x\right)\approx \frac{x}{{\bar{\gamma }}_{RD}}. \end{array}$$

Then, substituting Equations (13) and (33) into Equation (27), we can obtain:

(34) $$\begin{array}{c} \mathrm{Pr}\left(\gamma_{D}\leq \gamma_{0}\right)\approx \sum_{i=1}^{\rho \left(A_{RP}\right)}\sum_{j=1}^{\tau_{i}\left(A_{RP}\right)}\frac{\chi_{i,j}\left(A_{RP}\right)}{{\bar{\gamma }}_{RD}}\left(D_{1}j\mu_{\langle i\rangle }+D_{2}\right). \end{array}$$

Furthermore, taking Equations (32) and (34) into Equation (24), the asymptotic expression of OP can be given by:

(35) $$\begin{array}{c} P_{out}^{\infty }\left(\gamma_{0}\right)\approx \\ \left\{\begin{array}{c} \sum_{\xi_{1}=0}^{m_{SP}-1}\cdots \sum_{\xi_{M}=0}^{m_{SP}-1}\frac{\alpha_{SR}\Xi \left(M\right)}{{\bar{\gamma }}_{SR}}\left[\frac{C_{1}\Lambda_{SP}!}{\Delta_{SP}^{\Lambda_{SP}+1}}+\frac{C_{2}\left(\Lambda_{SP}-1\right)!}{\Delta_{SP}^{\Lambda_{SP}}}\right]+\sum_{i=1}^{\rho \left(A_{RP}\right)}\sum_{j=1}^{\tau_{i}\left(A_{RP}\right)}\frac{\chi_{i,j}\left(A_{RP}\right)}{{\bar{\gamma }}_{RD}}\left(D_{1}j\mu_{\langle i\rangle }+D_{2}\right)\\ -\sum_{\xi_{1}=0}^{m_{SP}-1}\cdots \sum_{\xi_{M}=0}^{m_{SP}-1}\frac{\alpha_{SR}\Xi \left(M\right)}{{\bar{\gamma }}_{SR}}\left[\frac{C_{1}\Lambda_{SP}!}{\Delta_{SP}^{\Lambda_{SP}+1}}+\frac{C_{2}\left(\Lambda_{SP}-1\right)!}{\Delta_{SP}^{\Lambda_{SP}}}\right]\\ \times \left[\sum_{i=1}^{\rho \left(A_{RP}\right)}\sum_{j=1}^{\tau_{i}\left(A_{RP}\right)}\frac{\chi_{i,j}\left(A_{RP}\right)}{{\bar{\gamma }}_{RD}}\left(D_{1}j\mu_{\langle i\rangle }+D_{2}\right)\right],\gamma_{0}<\min\left(\frac{1}{k_{1}^{2}},\frac{1}{k_{2}^{2}}\right)\\ 1,\gamma_{0}\geq \min\left(\frac{1}{k_{1}^{2}},\frac{1}{k_{2}^{2}}\right). \end{array}\right. \end{array}$$

Finally, denoting ${\bar{\gamma }}_{SR}={\bar{\gamma }}_{SP}={\bar{\gamma }}_{RD}={\bar{\gamma }}_{RP}=\bar{\gamma }$ and ignoring the higher order terms, we can get:

(36) $$\begin{array}{c} P_{out}^{\infty }\left(\gamma_{0}\right)=\Phi {\left(\frac{1}{\bar{\gamma }}\right)}^{\Theta }, \end{array}$$

where the diversity order Θ and coding gain Φ can be, respectively, derived as:

(37) $$\begin{array}{c} \Theta =\left\{\begin{array}{c} \Theta_{1}=1+\sum_{\xi_{1}=0}^{m_{SP}-1}\cdots \sum_{\xi_{M}=0}^{m_{SP}-1}\Lambda_{SP},\Phi_{1}>\Phi_{2},\\ \Theta_{2}=1,\Phi_{1}\leq \Phi_{2}, \end{array}\right. \end{array}$$

(38) $$\begin{array}{c} \Phi =\left\{\begin{array}{c} \Phi_{1}=\sum_{\xi_{1}=0}^{m_{SP}-1}\cdots \sum_{\xi_{M}=0}^{m_{SP}-1}\alpha_{SR}\Xi \left(M\right)\\ \times \left[\frac{C_{1}\Lambda_{SP}!}{{\left(\beta_{SP}-\delta_{SP}\right)}^{\Lambda_{SP}+1}{\bar{\gamma }}_{SP}}+\frac{C_{2}\left(\Lambda_{SP}-1\right)!}{{\left(\beta_{SP}-\delta_{SP}\right)}^{\Lambda_{SP}}}\right],\\ \Phi_{2}=\frac{D_{2}}{{\bar{\gamma }}_{RD}}. \end{array}\right. \end{array}$$

### 3.4. The Throughput of the System

It is very essential for us to analyze the throughput for the system, especially the terrestrial user *D*. According to [18], the definition of throughput for two time slot networks can be expressed as:

(39) $$\begin{array}{c} T=\frac{R_{s}}{2}\times \left[1-P_{out}\left(\gamma_{0}\right)\right]. \end{array}$$

where $R_{s}$ is the target rate of the system.

By substituting the analytical and asymptotic OP expressions into Equation (39), the analytical and the asymptotic expressions of throughput are derived. In order to reduce the length of the paper; hence, we do not give the final expressions here.

## 4. Numerical Results

Numerical Monte Carlo (MC) simulation results are presented to show the correctness of our analytical results. With no loss of generality, we assumed $\delta_{R}^{2}=\delta_{D}^{2}=1$, and through the whole figures we instructed ${\bar{\gamma }}_{SR}={\bar{\gamma }}_{SP}={\bar{\gamma }}_{RD}={\bar{\gamma }}_{RP}=\bar{\gamma }$, $k_{1}=k_{2}=k_{SP}=k_{RP}=k$, $T_{SR}=T_{RD}=T_{SP}=T_{RP}=L$ and $R_{s}$ = 10 bit/s/Hz, $U\in \left\{SR,SP\right\}$. The system and channel fading parameters are presented in Table 1 [22] and Table 2 [35], respectively.

**Figure 2:**
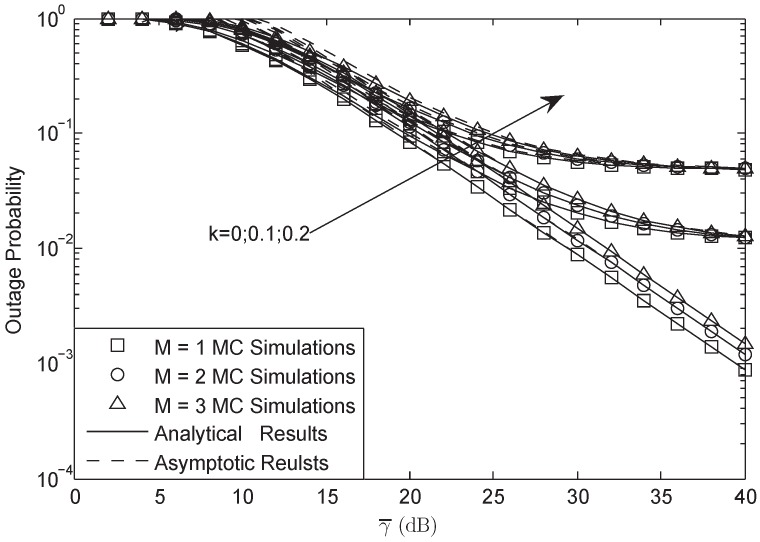
OP of the system versus different $\bar{\gamma }$ with σ=0.8, L=10 and $\gamma_{0}$ = 3 dB: FHS.

**Figure 3:**
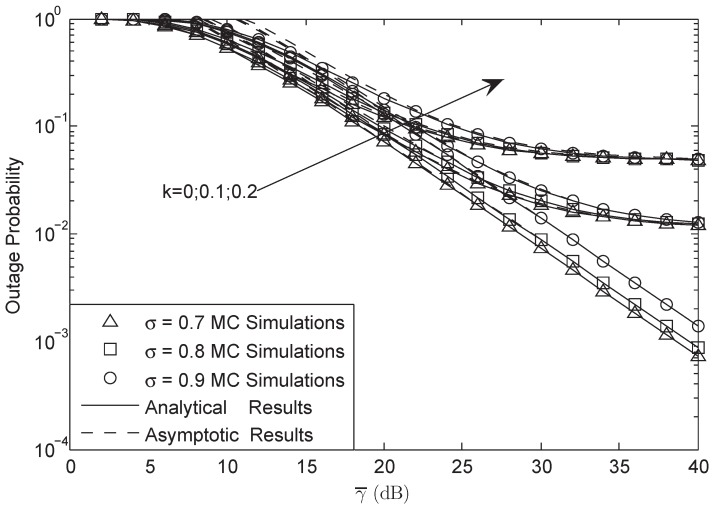
OP of the system versus different $\bar{\gamma }$ with M=1, L=10 and $\gamma_{0}$ = 3 dB: FHS.

Figure 2 and Figure 3 illustrate the OP of the system versus different $\bar{\gamma }$ with L=10 and $\gamma_{0}$ = 3 dB in FHS. As presented in both figures, the analytical results were in accurate agreement with MC simulations; besides, the asymptotic plots matched very well with the exact plots at high SNRs, which verifies the correctness of our derivation. They also showed that HIs and CEEs affect the system performance to a much higher degree at higher SNRs than that of lower SNRs. Furthermore, it was found that at high SNRs, HIs played the main role, while at low SNRs, the CEEs were the dominant factor. In addition, when the system suffered from HIs, the OP at high SNRs would have a lower bound. The larger the HIs level was, the larger the bound was. Besides, the OP would be decreased with the decreasing of *M* and σ; this is because with the decrease of *M* and σ, the power of the secondary user would be enhanced, which would lead to a lower OP.

**Figure 4:**
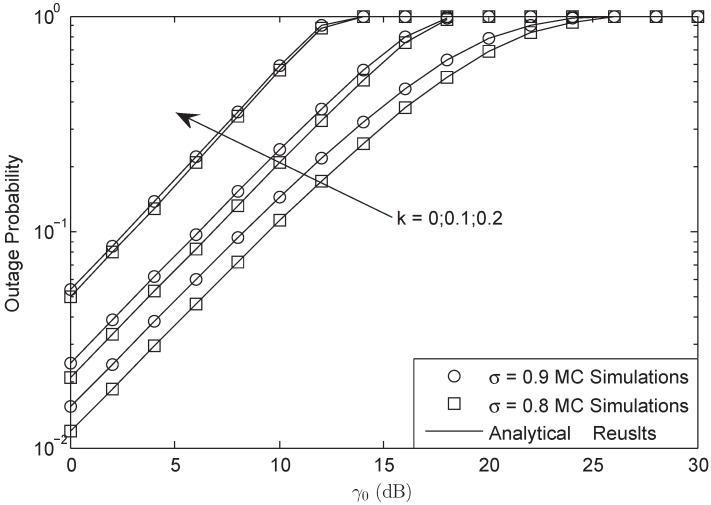
OP of the system versus different $\gamma_{0}$ with M=3, $\bar{\gamma }$ = 30 dB and L=10: FHS.

**Figure 5:**
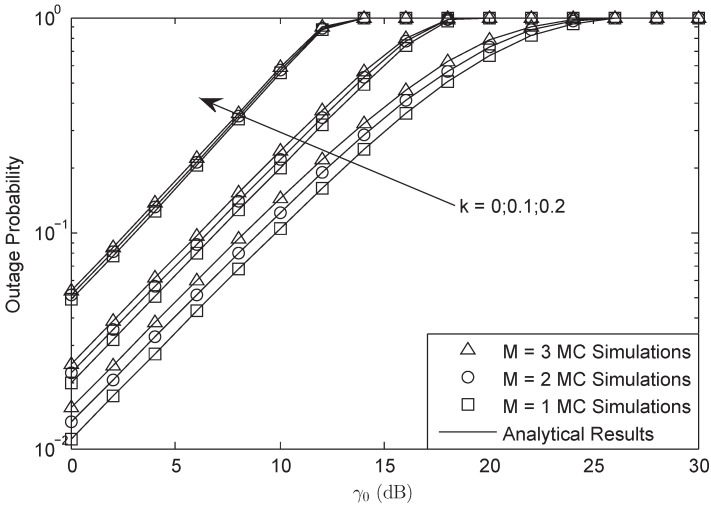
OP of the system versus different $\gamma_{0}$ with σ=0.8, $\bar{\gamma }$ = 30 dB and L=10: FHS.

In Figure 4 and Figure 5, OP is plotted against different $\gamma_{0}$ with $\bar{\gamma }$ = 30 dB and L=10 in FHS. We found that there was an SNDER ceiling for the OP plots, which means that there existed a special average SNR value that corresponded to the largest SNDER of the HI system. In other words, there was a fixed SNDER threshold beyond which the OP value always remained one, which has been proven in Equation (30). Moreover, we observe that different *M* systems will have the same ceilings and that different σ systems have the same ceilings as well. The aggregate level of HIs affects the ceiling effect all on its own.

**Figure 6:**
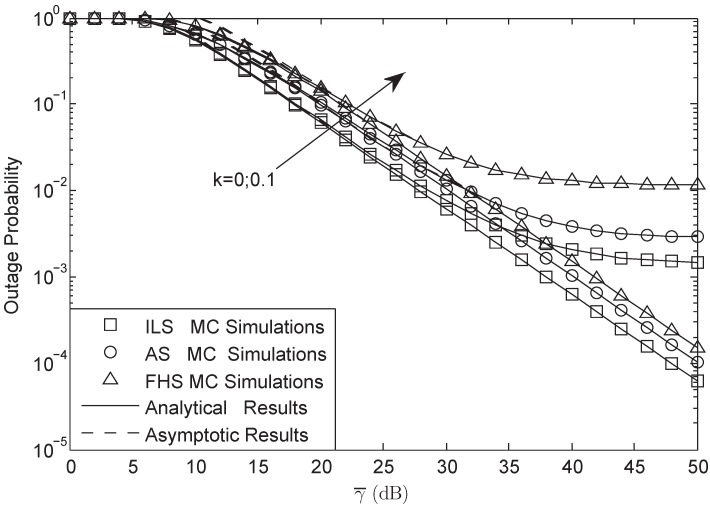
OP of the system versus different $\bar{\gamma }$ with M=3, σ=0.8, $\gamma_{0}$ = 3 dB and *L* = 10.

Figure 6 plots the OP of the system versus different $\bar{\gamma }$ with *M* = 3, σ = 0.8, $\gamma_{0}$ = 3 dB and *L* = 10. We can obtain that the OP will be larger when the channel was under heavy fading. Moreover, we also found that the OP would be higher when the system was under HIs.

**Figure 7:**
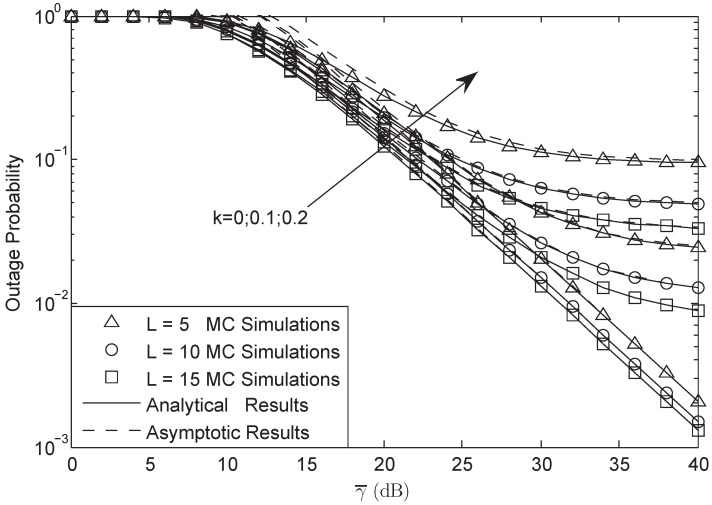
OP of the system versus different *L* with M=3, σ=0.8 and $\gamma_{0}$ = 3 dB.

Figure 7 examines the OP of the system versus the training symbol length *L* with *M* = 3, $\gamma_{0}$ = 3 dB and σ = 0.8. We can observe that the OP would be lower with the length of pilot symbol increasing as a result of that when *L* was larger, the CSI of the channel would be more accurate. The more accurate CSI would lead to the better system performance.

**Figure 8:**
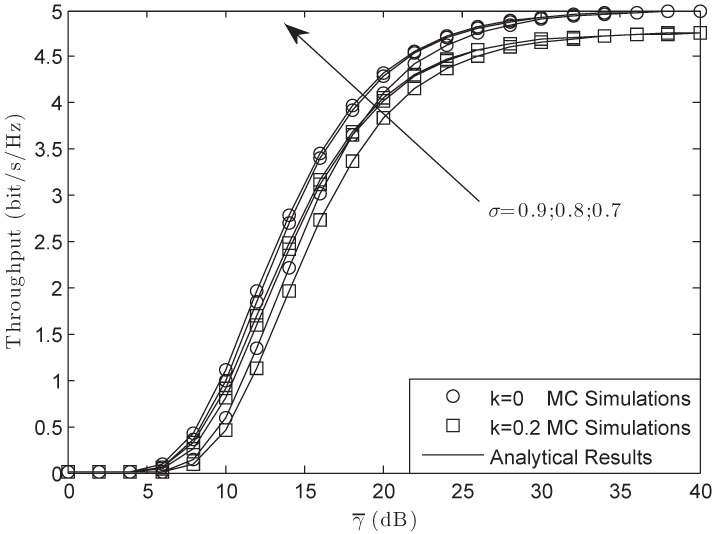
Throughput of the system versus different $\bar{\gamma }$ with *M* = 3, *L* = 10 and $\gamma_{0}$ = 3 dB: FHS.

Figure 8 illustrates the throughput of the system versus different $\bar{\gamma }$ with *M* = 3, *L* = 10 and $\gamma_{0}$ = 3 dB in the FHS condition. From the figure, we observe that the throughput was lower than that of the target rate $R_{s}$ for the reason that the system suffered HIs. Furthermore, from this figure, the system would have larger throughput when σ was smaller as the result of the power for the pilot signal being larger. However, we find that when the hardware of the system was ideal, the throughput of the system would be $R_{s}/2$ as the SNR of the system became larger enough.

**Figure 9:**
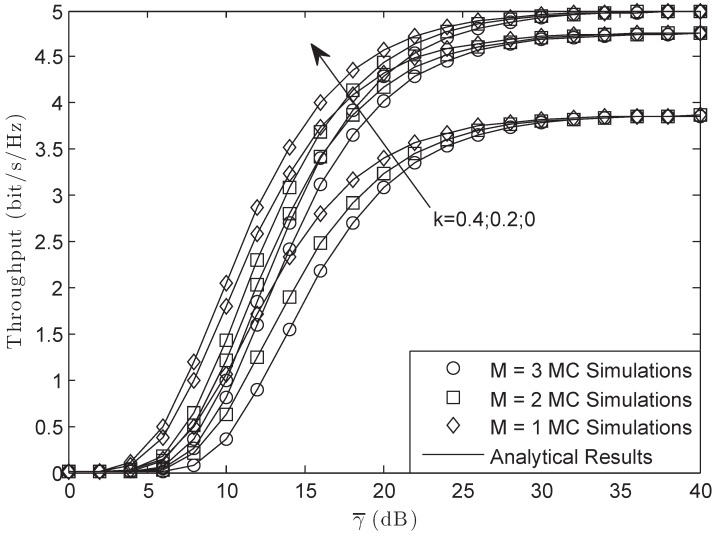
Throughput of the system versus different $\bar{\gamma }$ with σ = 0.8, *L* = 10 and $\gamma_{0}$ = 3 dB: FHS.

Figure 9 examines the throughput of the system versus different $\bar{\gamma }$ with σ = 0.8, *L* = 10 and $\gamma_{0}$ = 3 dB in the FHS condition. From the figure, we can obtain that the throughput would be lower with the increase of the number for PUs. This is because when the number of the PUs was larger, the power of the secondary user would be smaller, which led to this scene. Besides, from the figure, we can also obtain that at high SNRs, the throughput would have a bound that was just the function of the impairments’ level. The larger the level was, the lower the bound was.

## 5. Discussion

In this paper, we have investigated the joint effects of CEEs and HIs on the performance of a CSTRN with multiple primary users. Specifically, the SNDER was first derived for the CSTRN by employing a general and practical model to account for both the CEEs and HIs. Moreover, the closed-form expressions for the OP and throughput have been obtained, which could be utilized to characterize the aggregate effects of CEEs and HIs. Furthermore, the asymptotic analysis of OP and throughput at high SNRs were also subsequently obtained, which gave a quantitative characterization for the impact of HI level and CSI imperfections on the considered network. We demonstrated that there was an upper bound on the SNDER when the system was in full outage. The OP and throughput would have a bound, respectively, when HIs exist. Moreover, we found that the bound was just the function of the HI level. Furthermore, we found that the improvement in the channel shadowing condition would enhance the system performance.

## Figures and Tables

**Table 1:** System parameters.

Parameters	Value
Satellite Orbit	GEO
Frequency Band	f=2 GHz
3-dB Angle	${\bar{\theta }}_{k}=0.8{}^{\circ }$
Maximal Beam Gain	$G_{max}=48$ dB
Antenna Gain	$G_{ES}=4$ dB

**Table 2:** Channel parameters.

Shadowing	$m_{U}$	$b_{U}$	$\Omega_{U}$
Frequent heavy shadowing (FHS)	1	0.063	0.0007
Average shadowing (AS)	5	0.251	0.279
Infrequent light shadowing (ILS)	10	0.158	1.29

## Abbreviations

The following abbreviations are used in this manuscript:

AS	average shadowing
AWGN	additive white Gaussian noise
CDF	cumulative distortion function
CEEs	channel estimation errors
CSI	channel state information
CSTRN	cognitive satellite-terrestrial relay networks
DF	decode-and-forward
EVM	error vector magnitude
FHS	frequent heavy shadowing
HIs	hardware impairments
HPA	high power amplifier
ILS	infrequent light shadowing
IQI	in-phase quadrature-phase imbalance
LOS	line of sight
MC	Monte Carlo
MMSE	minimum mean square error
OP	outage probability
PDF	probability distribution function
PN	phase noise
PUs	primary users
SER	symbol error rate
SNDER	signal-to-noise-plus-distortion-and-error ratio
SNR	signal-to-noise-ratio
SR	shadowed Rician
STRN	satellite-terrestrial relay network
